# Genetic pattern and gene localization of polydactyly in Beijing fatty chicken

**DOI:** 10.1371/journal.pone.0176113

**Published:** 2017-05-10

**Authors:** Chuan He, Yongcan Chen, Kaixuan Yang, Zhengxiao Zhai, Wenjing Zhao, Shuyun Liu, Jinmei Ding, Ronghua Dai, Lingyu Yang, Ke Xu, Zhenxiang Zhou, Caiju Gu, Qizhong Huang, He Meng

**Affiliations:** 1 Department of Animal Science, School of Agriculture and Biology, Shanghai Jiao Tong University; Shanghai Key Laboratory of Veterinary Biotechnology, Shanghai, China; 2 Animal Husbandry and Veterinary Research Institute, Shanghai Academy of Agricultural Science, Shanghai, China; Xiamen University, CHINA

## Abstract

Polydactyly, a common heritable limb malformation in vertebrates, is characterized by supernumerary digits. In chickens, basic characteristics and rough dominant genes have been explored in past decades; however, the elaborate pattern of inheritance and the determinant gene remain obscure. In this study, different types of polydactylism were classified by the numbers and the shapes of toes, including the newly defined subtypes of B’ and G, for the Beijing fatty chicken, a native breed of chicken from China. Through experiments on hybridization, we demonstrated a complete dominant inheritance of polydactyly instead of an incomplete penetrance or genetic modification of the previous conjecture. In particular, by using the F2 population of the five-digit purebred line of Beijing fatty chicken backcrossed to Shiqiza chicken and by using restriction-site associated DNA based markers, we performed a genome-wide association study on the trait of polydactyly. Furthermore, whole genome resequencing strategy was applied to sweep SNPs across the whole genome. An outlier-based F_st_ approach was employed to search for signatures of selection, and results indicated that the determinant mutation was found in the region ranging from 8.3 Mb to 8.7 Mb, where the polydactyly candidate gene LMBR1 was located. The G/T mutation of rs80659072 was identified to be highly associated with polydactyly in our resequencing and was validated in random samples from an expanded population. Thus, we confirmed that LMBR1 was the causative gene of polydactyly in the Beijing fatty chicken by using GWAS with restriction-site associated DNA based markers and resequencing.

## Introduction

Polydactyly (Po) is a common heritable limb malformation in numerous vertebrates and is characterized by supernumerary digits [1–4]. In chickens, the usual number of digits is four, which makes the phenotype of Po in chickens to manifest as an extra toe on one or both feet. These additional toes are the result of the development of new toes on the opposite side of the foot rather than the restoration of the fifth digit that has been lost from the typical pentadactyl foot [5, 6]. The phenotype of five toes constitute a characteristic in some famous breeds of chicken.

It is generally thought that the mutation of Po in fowls is caused by an incomplete penetrant autosomal-dominant gene [7–9]. By using bulked segregant analysis (BSA) strategy, Pitel et al. mapped the candidate gene close to microsatellite marker MCW0071 on chromosome 2p [10], which had an area homologous to human and mouse autosomes that had been proven to regulate limb development [11]. Our research group had performed a linkage analysis with microsatellites and thus mapped the Po locus to the region between ABR0004 and MCE0184 of chromosome 2p [12]. Huang et al. reported that one mutation (T/C) in exon 13 of an LMBR1 clone was associated with Po by performing single strand conformation polymorphism (SSCP) in a hybrid F_2_ population of the cross between Silkies and broilers [13]. Furthermore, recent studies on Po have further mapped the causative variant to several candidate genes in chromosome 2 through linkage and GWAS with SNPs identified by arrays [14, 15].

The Beijing fatty chicken (BF) is an excellent Chinese indigenous breed with some distinctive features such as combs that are covered by heavy feathers, feathered yellow legs and polydactyly. According to statistics, there is an incidence of Po ranging from 25% to 31% in the natural population of BF [16]. As a very popular breed on the Chinese market for its flavor, it is profitable to fix the trait of Po in BF as an observable carcass trait of dressed chicken in local market. In addition, owing to its morphological variations, BF can be an ideal model to study Po and other features in fowls.

The spectrum of genetic variants across the whole genome can be applied to various population genetics studies. As a complementary procedure of whole genome resequencing, reduced-representation genome sequencing has turned out to be widely suitable for plants and animals [17,18]. Compared to resequencing, reduced-representation genome sequencing is more economical and efficient. Among them, restriction-site-associated DNA sequencing (RAD) can be cost-effective and can produce high coverage orthologous markers. Considering its relatively higher tag density in the genome than other procedures of reduced-representation genome sequencing for a given restriction enzyme, RAD was applied to our previous research in Chinese indigenous breeds of chickens [19].

To perform genetic analysis and gene mapping, we established a purebred polydactylous BF line over the past decade. In this study, GWAS using RAD-based markers was applied to a testcross of our purebred polydactylous BF purebred line to normal Shiqiza (SZ) that was bred for three generations in hopes of finding the determined gene of Po. To validate the SNPs found by RAD and to scan all of the mutations linked to the markers associated with the trait of polydactyly, whole genome re-sequencing was performed.

## Results

### Classification of polydactylism

**Fig 1:**
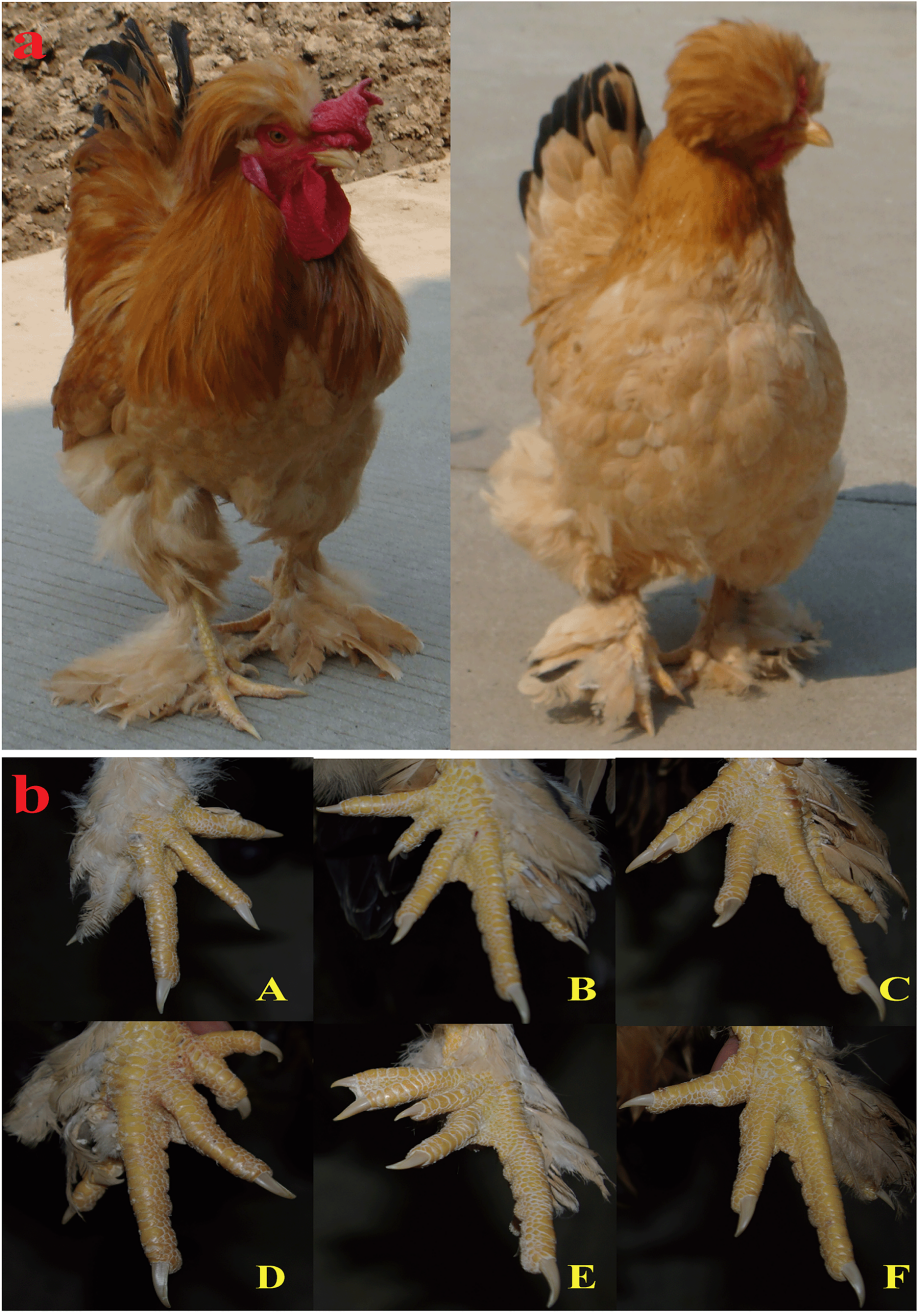
Polydactyly subtypes of Beijing fatty chicken. (a) Beijing Fatty rooster and hen. (b) Warren’s classification of polydactyly in chicken. The letters in red correspond to different subtypes.

In Europe, Po was considered to be originally from Silkie and Houdan chickens and present in various crossbred stocks. In 1943, Warren described and classified 5 major types of polydactylism of the feet [5]. As Fig 1B shows, type A is called polyphalangy and refers to a reversion of the four-toed condition in which the inner toe has an extra phalanx. Type B, the most usual form of polydactylism, is characterized by an extra long digit that grows at the inside base of the first toe. Type C may be considered to be type A with a long inside toe split. Type D possesses a short extra toe. Type E has six toes. Type F is a result of when types A and C show a vestige of the hallux in the positions where the digit occurs in types B and E.

All subtypes of the trait of Po described above were found with similar incidence in our pilot experiments with purebred and crossbred birds. Furthermore, several new variants were found and were named type G, A’ and B’ according to their respective morphologies. As S1 Fig shows, type G has the same number of toes as type E, but the two extra digits are shorter. Type A’ and B’ are, respectively, variants of type A and B with an additional digit growing from the extra phalange instead of from the inside base of the first toe. Based on observations (S1 Table), types B and D account for the majority of the polydactylous BF population.

### Purebred and crossbred in polydactylous BF

Results of reciprocal mating between homogenous polydactylous BF and SZ / Yellow Dwarf (YD) are shown in S2 Table. Sex-link inheritance was eliminated because the penetrance was similar regardless of whether the Po gene was inherited from male or female chickens; these results agreed with former reports [5–9]. In addition, the fact that nearly the same incidence of polydactyly was present in different cross combinations demonstrated that the Po candidate gene was dominant across many strains of indigenous chickens.

Nine typical type B (as described by Warren) roosters, a group which consisted of 5 individuals with the highest penetrance in the pilot (G_0_) and 4 of their sons, were selected to be male parents. These roosters were mated to the daughters of G_0_, and their progeny were then classified as the first purebred generation (G_1_). The number of toes of G_1_ was recorded and is shown in S3 Table. According to the results of the testcross to SZ, 11 roosters and 40 hens from the second generation, which showed the penetrance of above 90% and 80%, respectively, were selected to mate, and thus generated 883 offspring. which were all polydactylous. The digit types of their progeny are summarized in S5 Table. The 100% incidence of Po suggested that it was a completely dominant trait.

### Localization of causative gene

HiSeq 2000 sequencing yielded a total of ~180 Gb raw data that passed Q20 filtering and covered approximately 700,000 RAD tags. These data accounted for an average of 11× sequencing depth per tag that was 8,254,916 reads per individual that met quality requirements. These reads were aligned to the reference genome (Gal 4.0) and a total of 767,810 high confidence SNPs were identified. As shown in S2 Fig, the distribution of high confidence SNPs across the chromosomes was relatively even. These high confidence SNPs were then input into PLINK [20], filtered with the rules listed in the methods, and yielded 476,239 SNPs.

**Fig 2:**
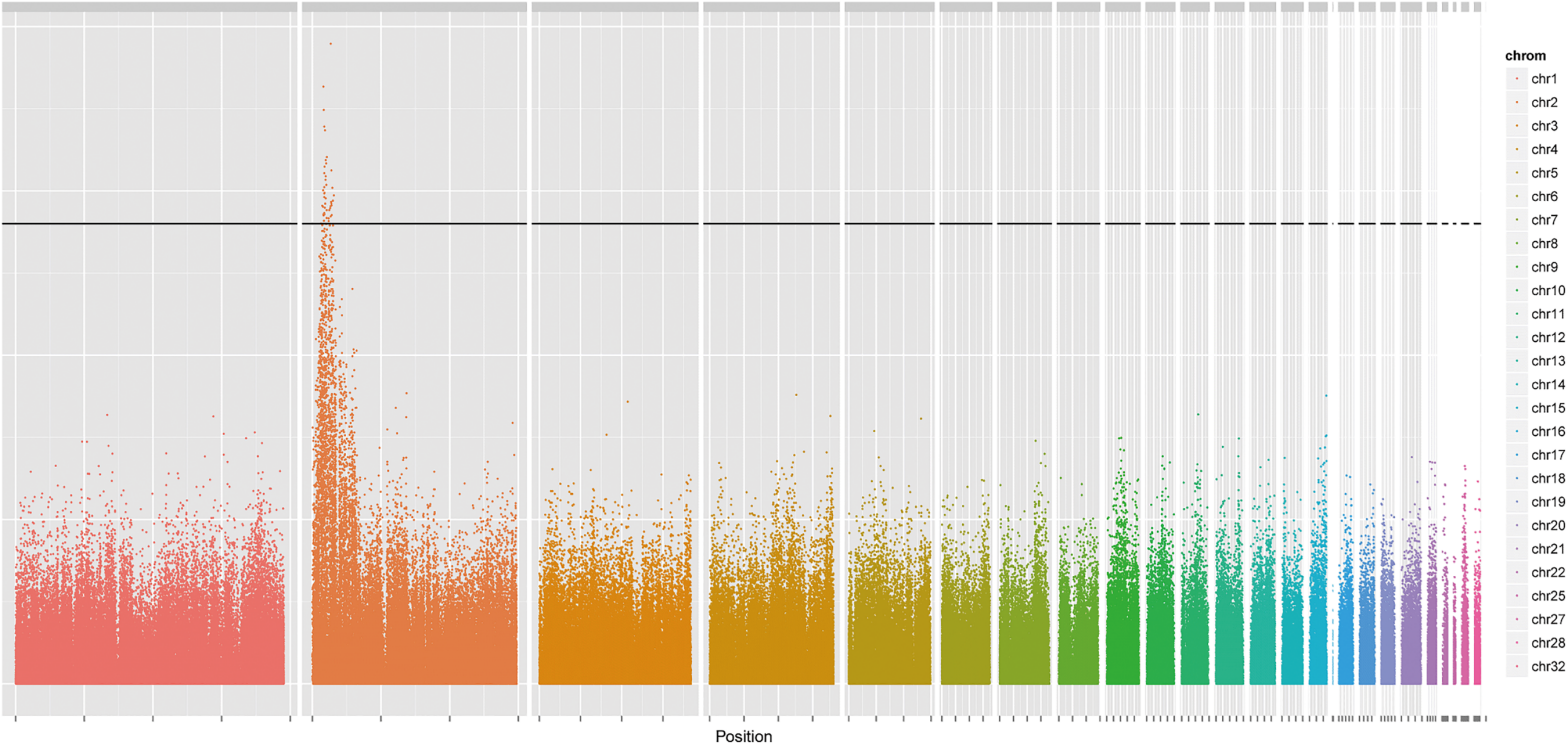
Genome-wide scan for polydactyly in the Beijing fatty chicken. Manhattan plot showing the association of all SNPs with the phenotype of polydactyly. Chromosomes 1–32 are shown in different colors. The black solid line indicates a significant association with a P-value approximately equal to 1.009e-07, a value with a Bonferroni correction below 0.05.

All 112 samples, consisting of 48 cases and 64 controls, were successfully genotyped. 35,578 independent SNP markers were included in multidimensional scaling (MDS) analysis. The first and second MDS components were used as covariates in the logistic model which we mentioned in the methods section. Meanwhile, a mixed linear model in a GCTA package [21] was applied to the subset of SNPs which passed the filtering in hopes of cross validating the association. The significances of all the SNP markers across the whole genome are shown in Fig 2. All significant SNPs revealed by both software programs are listed in Table 1 with their nearest gene.

**Table 1:** SNPs significantly associated with the polydactyly phenotype.

chr	position	A1/A2	OR	mlm P ^a^	logistic P ^b^	nearest gene	distance ^c^
2	6306869	T/C	76.25	3.44E-11	9.97E-08	PRKAG2	0
2	7097528	A/G	0.03146	9.89E-10	1.29E-07	ACTR3B	282867
2	7978373	C/T	52.9	4.30E-10	3.48E-07	RBM33	0
2	8003962	C/T	55.69	3.83E-12	8.20E-10	RBM33	0
2	8263544	A/G	35.46	1.46E-10	1.86E-09	RNF32	150902
2	8645181	C/A	37.82	3.09E-11	3.31E-09	DNAJB6	14950
2	8678907	C/A	74.67	3.88E-11	1.32E-07	DNAJB6	0
2	8909242	G/C	40.56	2.27E-10	3.41E-08	PTPRN2	0
2	8909250	C/T	40.56	2.27E-10	3.41E-08	PTPRN2	0
2	9251806	T/C	23.14	8.59E-10	3.78E-09	PTPRN2	0
2	10070358	G/A	31.95	2.01E-10	1.20E-08	DIP2C	70587
2	10472227	C/A	30.86	2.64E-10	9.68E-09	ADARB2	0
2	13408892	A/T	41.71	4.03E-12	1.83E-10	PARD3	0
2	13408966	A/G	41.71	4.03E-12	1.83E-10	PARD3	0
2	14923383	G/A	22.44	8.93E-10	4.47E-08	MTPAP	0

a mlm P stands for P value calculated by mixed linear model.

b logistic P stands for P value calculated by logistic model.

c distance column shows the distance between the significant SNPs and their nearest genes, either 3’ or 5’ flank.

The most significant SNP was located at 1340.88 kb (P = 1.83 × 10^−10^) in the intron 22 of the partitioning defective 3 homolog gene (PARD3); this gene is known to be essential for asymmetric cell division and polarized growth [22]. There was a significant SNP located (P = 8.20 × 10^−10^) at 76 kb downstream and 430 kb upstream of, respectively, the sonic hedgehog gene (SHH) and the limb region 1 homolog (LMBR1) [23–25], two of the most important candidate genes for Po. In addition, other highly significant SNPs were located near the RING finger protein 32 (RNF32), DNAJ homolog subfamily B member 6 (DNAJB6), condensin-2 complex subunit G2 (NCAPG2) and double-stranded RNA-specific editase B2 (ADARB2), which were all located in the region between 8 Mb to 13.4 Mb and had never been reported to be associated with the trait of Po.

### Comparison of whole genome sequences

A total of ~200 Gb raw reads were generated by sequencing six samples, with a theoretical 30-fold coverage for each sample compared with the expected genome size. 90% of the whole genome assembly was covered at least 20-fold depth. 9,896,366 high confidence SNPs were found, and 6,079 of these found SNPs were identified to be opposite homozygous genotypes between polydactylous and normal samples. Being opposite homozygotes indicates that the genotype at a specific allele is homozygous and in the same intra-population but different inter-population, the number of which should be generally positively correlated with the length of the chromosome. However, 2,831 opposite homozygotes were from chromosome 2, approximately two times of the quantity in chromosome 1, although the latter had a longer physical length. The same situation occurred to the InDels. 693 of 1,322,701 InDels were opposite homozygotes and 355 came from chromosome 2. It was interesting that, although consisting of the most opposite homozygous SNPs and InDels, chromosome 2 was not the densest when compared with other chromosomes. 1,057 opposite homozygous SNPs and 100 opposite homozygous InDels were found in chromosome 9, which gave it the highest density among all the chromosomes.

**Fig 3:**
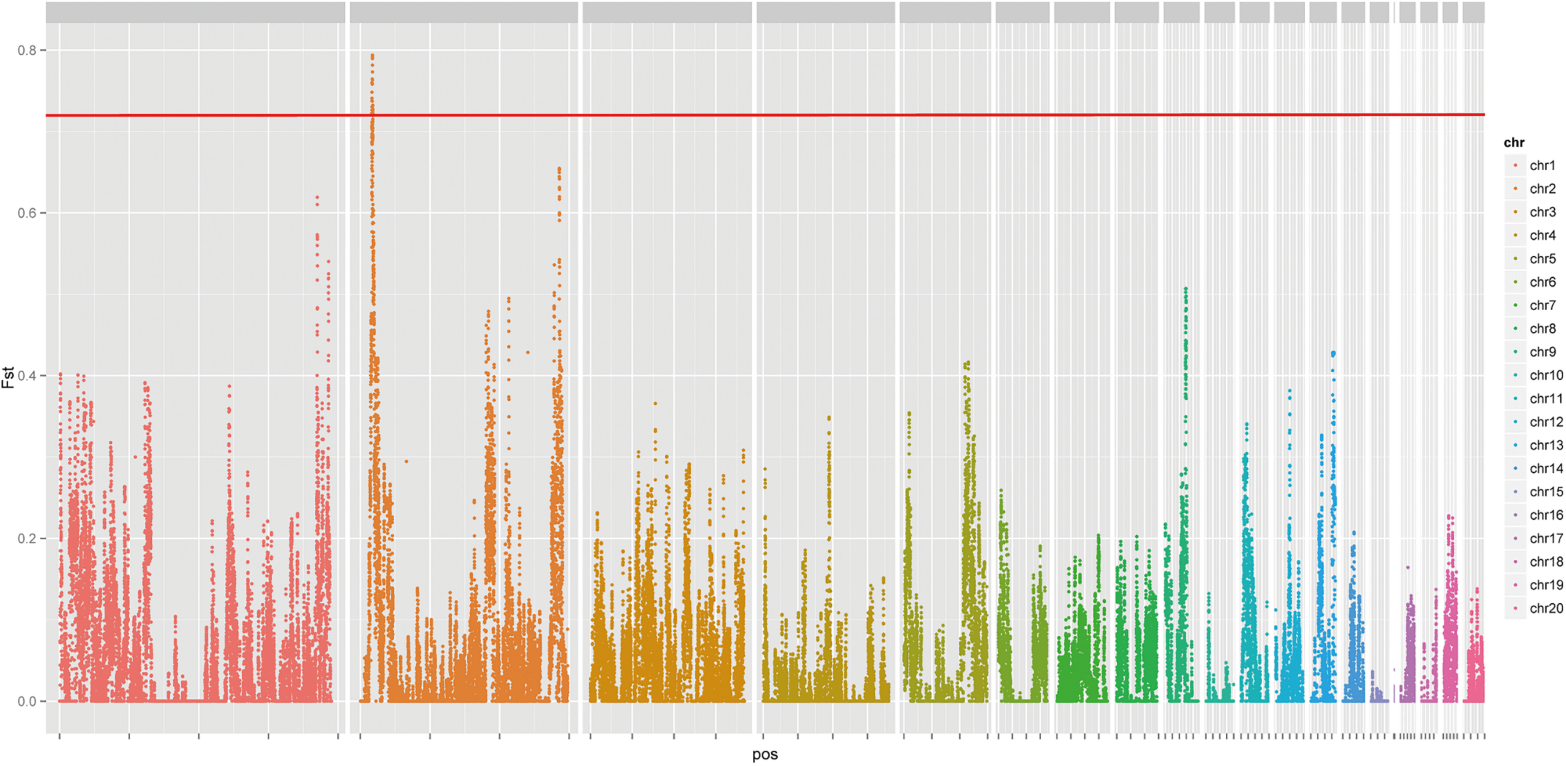
Distribution of pairwise F_st_ across the whole genome. Chromosomes 1–20 are shown in different colors in the Manhattan plot. The red solid line is y = 0.72, corresponding to the lower bound of the highest 0.1% F_st_ value.

The signatures of artificial selection can be reflected in allele frequencies in a long range and is known as the hitch-hiking effect [26]. This effect assumes that the artificially selected region possesses the most differentiated SNPs, resulting in outlier genetic differentiation (F_st_) [27]. Thus, to detect the specific genomic region most likely to have experienced high strength artificial selection, an outlier-based approach based on 100 kb sliding windows with a step size of 10 kb was employed. As shown in Fig 3, the highest F_st_ value exceeded the 99.9% level of the empirical distribution gathered in the region that ranged from 8.3 Mb to 8.7 Mb, where LMBR1 was located. Various sliding window-size and step-size resulted in similar areas.

It was worth noting that the gene region of LMBR1 (8,434,744–8,494,298 bp) precisely matched the overlap region of a GWAS significant locus and outlier F_st_. Given former studies on chicken polydactyly, LMBR1 deserves complete attention. 79 opposite homozygous SNPs were located in the region of LMBR1; one of these SNPs, rs80659072, was strongly associated with the trait of Po in Silkie chickens [23–25]. In particular, in the range of the highly important intron 5 which was previous reported, rs80659072 was the only opposite homozygous SNP.

### Verification of mutations in the expanded population

To further validate the SNPs that were identified by resequencing, Sanger sequencing was applied to some of the promising candidate opposite homozygous SNPs in 138 randomly selected samples. As Table 2 shows, rs80659072 was the most strongly associated SNP with Po (results of the other SNPs were not listed). Every individual with an allele of T in loci rs80659072 turned out to be Po. This result suggested that rs80659072 was, if not the causative variant, strongly linked to polydactyly, which confirmed our conclusion from GWAS and resequencing.

**Table 2:** Genotype of rs80659072 in validation group.

	Genotype		
Phenotype	GG	GT	TT
Polydactyly	0	39	52
Normal	45	2	0

## Discussion

As far back as 1920, Po was demonstrated to be autosomal dominant to the normal four-digit trait in fowls [11] and the determinant gene was mapped to the forth linkage group [28]. However, homozygous polydactylous parents have occasionally given birth to normal progeny, which has resulted in the hypothesis that genetic modifiers [29] and environmental factors [30] and may interfere with the trait’s penetrance. To eliminate the interference of various forms of polydactylism in our pure breeding procedure, we strictly selected for type B sires and dams to mate and consequently obtained a purebred line with 100% penetrance of polydactyly. The cross of the purebred polydactylous BF line to SZ demonstrated that Po was a complete dominant trait even though it should be noted that a complete penetrance did not result in 100% type B polydactylism.

Former efforts in Po gene mapping in exploited Silkie or normal BF chickens had resulted in a relatively low incidence of Po, which always led to lack of positive cases for sufficient statistical power. In addition, types other than B might be inherited in a more complex manner [5] and, thus, might convolute the results of gene mapping. With the help of our purebred polydactylous BF line, we successfully applied gene mapping with SSRs in 2013 [12] and RAD-seq in the current study. We thus proved that our purebred line would be a good model for research on avian Po.

Our work provided a new integrated strategy for gene mapping. First, RAD was proven to be an effective tool for genotyping and population genetic studies [31–33]. In this research, RAD generated adequate and evenly distributed SNP markers of high confidence for GWAS. The fact that all significant SNPs associated with polydactyly were located in the 6–13.4 Mbp region of chromosome 2 was concordant with previous studies, suggesting that RAD could be utilized for avian GWAS. However, one of the drawbacks of reduced representation sequencing is its limited coverage of the whole genome. Thus, whole-genome resequencing was also applied to sweep the variants located near significant markers in an effort to uncover as many variations as possible. SNPs in the regions which have been under strong artificial selection would be purified [34, 35]. The abnormal level of homozygosity in chromosomes 2 and 9 suggested some regions under positive selective pressure; however, the F_st_ outlier method ruled out chromosome 9 because of a relatively even distribution of opposite homozygous SNPs. Comprehensive implication of RAD and whole genome scanning can be a conventional procedure of gene mapping.

Previous studies in mice and humans have shown that LMBR1 and SHH play a critical role in Po [36–38]. Intron5 of LMBR1 has been named the “zone of polarising activity regulatory Sequence” (ZRS) [23] and was considered to be the cis-regulatory region of the Sonic hedgehog (SHH) gene. Another study on Silkie chickens revealed a strong association of the same SNP ss161109890 (rs80659072 in dbSNP) with Po [25] in a manner that was concordant with the dominant and incomplete penetrant nature of this trait. Dunn et al. further demonstrated that the genotype of rs80659072 was strongly associated with the ectopic expression of SHH, which led to preaxial Po [24]. In this paper, we demonstrated that SNP rs80659072 showed highly association with the trait of Po in BF and was dominant in several Chinese indigenous strains; these results agreed with the latest research [39].

In conclusion, a highly purebred BF line with 100% penetrance of Po was obtained and served as an ideal model for the study of Po in fowls. By observing the process of pure breeding and cross breeding, we provided evidence that chicken polydactyly was a complete dominant trait. With the help of an integrated strategy of RAD-seq and resequencing, we further confirmed that this dominant trait was highly associated with the genotype of rs80659072 in the BF line.

## Materials and methods

### Purebred and crossbred in polydactylous BF

First, a pilot experiment including the pure breeding of polydactylous BF and the crossbreeding of BF and SZ was performed. Observation lasted for five generations, and all types of toe-characteristics from both feet were recorded. A reciprocal cross between BF and SZ / YD was then performed. Ten typical type B polydactylous BF roosters from the pilot experiment were selected, and each rooster was mated to 3 normal BF hens, 3 SZ hens and 3 YD hens. Meanwhile, 10 SZ roosters and 10 YD roosters were mated to polydactylous BF hens at the same mating ratio.

Due to the high penetrance of Po in their progeny, 5 polydactylous BF roosters and 4 of their sons (foot labels shown in S4 Table) were selected to mate with their daughters. A testcross of the second generation (G_1_) to SZ was applied to confirm the five-digit progeny’s genotypes. Roosters and hens from G_1_ with a penetrance of above 90% and 80%, respectively, were selected to mate, and generated 882 polydactylous progeny (G_2_) which were more than 95% typical type B. Since then, the pure bred polydactylous BF population have been reproductively isolated by mating roosters and hens with top penetrance. All the birds were bred indoors at the research base in Animal Husbandry and Veterinary Research Institute, Shanghai Academy of Agricultural Science, Shanghai, China, under standardized conditions.

### Animals and RAD sequencing

All animal experiments followed the guidelines established by the ethics committee for the Care and Use of Laboratory Animals in Shanghai Jiao Tong University. The protocol was approved and the permit number is 2008–0713). A three-generation design was used by crossing male purebred polydactyly BF and female SZ. The F_2_ resource population was derived from backcross of male F_1_ hybrid chickens to female SZ. A total of 112 individuals, consisting of 11 families, from this population (including 1 male and 1 female progenitor from P, 1 sire from F_1_ and 11 wild type dams, 98 offspring from F_2_) were sampled. The number of digits on their feet was recorded. The pedigree of all individuals and their digit numbers are shown in Supporting Information, S4 Table. Genomic DNA was extracted following the standard phenol/chloroform method. With HindIII digestion, the constructed RAD tag libraries were sequenced by Illumina HiSeq (a sequencing service provided by Personal Biotechnology Co., Ltd. Shanghai, China).

#### GWAS

The next generation sequencing reads were aligned to the reference genome of *Gallus gallus* (galGal4) with BWA v0.7.12 [40] in order to identify SNPs. High confidence SNPs were called by a mixed use of GATK [41] and SAMtools v0.1.18 [42]. The SAMtools output SNPs were transformed into PED and MAP files, which were the standard input files of PLINK-1.07. SNPs were further discarded under the following conditions: (1) the minor allele frequency (MAF) < 0.05; (2) single SNP missing rate across all individuals > 0.5; and (3) the P-value of Hardy-Weinberg equilibrium (HWE) test < 10–6. The population structure was assessed by MDS analysis based on the identity-by-state (IBS) matrix.

GWAS was performed with PLINK by using a logistic model with the first and second MDS components as covariates. The significant values were adjusted with a Bonferroni correction. A mixed linear model was applied to the same subset of SNPs with a GCTA package.

### Whole-genome resequencing

DNA from six BF males (three polydactylous and three normal ones), all presumed to be homozygous for each phenotype, were extracted. Six sequencing libraries were generated and sequenced for each sample with the standard NextSeq 500 protocol. Quality control was performed with our in-house Perl script; the adaptors were trimmed and reads with average quality above 20 were retained. Trimmed reads which passed QC were aligned to the galGal4 genome with BWA v0.7.12. Picard 1.107 was performed to remove PCR duplicates, and GATK was then used to call high quality SNPs and InDels. F_st_ between the polydactylous and normal population was calculated with in-house Perl scripts by using a sliding-window approach.

### Validation of candidate mutation

Blood was sampled from individuals that were randomly selected from the natural population of BF. Genomic DNA was extracted following the standard phenol/chloroform method. PCR steps were 94°C, 5 min for 1 cycle, 94°C, 30 sec, 60°C, 30 sec, 72°C, and 30 sec for 35 cycles. The primers of rs80659072 were: F: 5’-CAGTGTAGCCCATCTCACCT-3’; R: 5’ -ACATGACAGCACAATGGAGGA-3’. The PCR products were sequenced by ABI 3730.

## Supporting information

S1 Fig New types of polydactylism in the Beijing fatty chicken. (TIF)

S2 Fig Distribution of high quality SNPs across the whole genome generated by RAD-seq. Each bar stands for a chromosome, whose length is proportional to its physical length (Mbp) in assembly of Gal4. The red tiles stand for the SNPs density in a 20000 bp region.
(TIFF)

S1 Table Summary of the digit types in the pilot experiment. (XLSX)

S2 Table Penetrance of polydactyly summary in the reciprocal cross experiment. Column 1 shows the roosters’ IDs. Column 2 shows the hens’ IDs. Columns 3 and 4 represent the number of wild-type and polydactyly progeny, respectively. Column 5 is the percentage of polydactyly progeny. Each group of cross is summarized in “sum” raws.
(XLSX)

S3 Table Summary of the second generation of pure breeding. 44 / 45 / 55 stand for, respectively, the count of progeny with different digit numbers on both feet.
(XLSX)

S4 Table Summary of the third generation of pure breeding. 56 / 66 / 45 / 55 stand for, respectively, the count of progeny with different digit numbers on both feet. A stands for type A of polydactylism.
(XLSX)

S5 Table Pedigree of the GWAS population and their phenotype. Column 1 shows the sample IDs. Columns 2 and 3 show the ID of sample’s dam and sire. The last column is the phenotype of the sample.
(XLSX)
