# Supplementary figures and images for: Genetic pattern and gene localization of polydactyly in Beijing fatty chicken
Source: PLoS One. 2017 May 10;12(5):e0176113. doi: 10.1371/journal.pone.0176113 (PMC5425009; doi:10.1371/journal.pone.0176113)

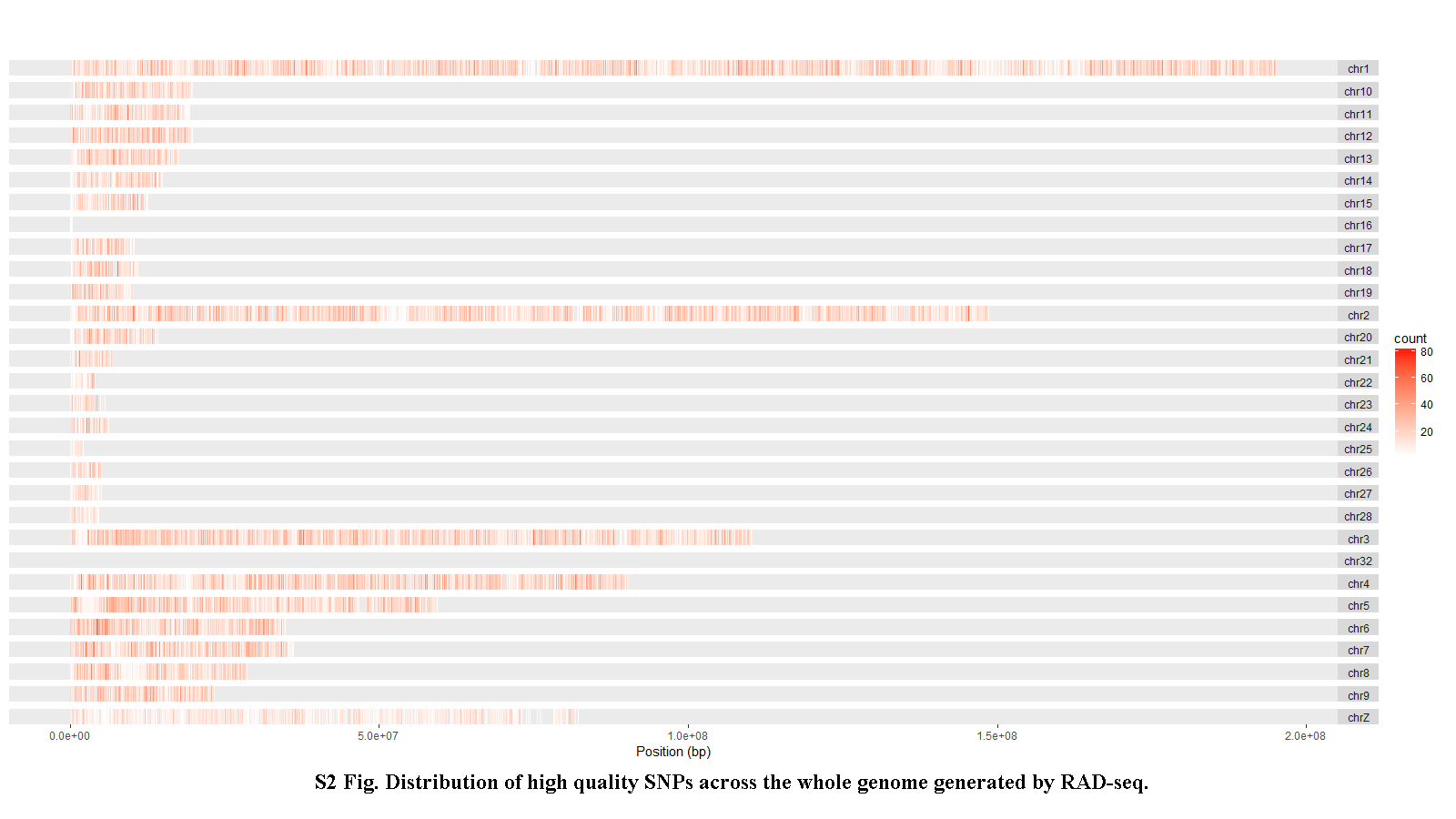

Supplement: S2 Fig — Each bar stands for a chromosome, whose length is proportional to its physical length (Mbp) in assembly of Gal4. The red tiles stand for the SNPs density in a 20000 bp region. (TIFF) [file pone.0176113.s002.tiff]
